# Prevalence and antimicrobial susceptibility pattern of toxigenic Clostridium difficile strains isolated in Iran

**DOI:** 10.3906/sag-1808-11

**Published:** 2019-02-11

**Authors:** Maryam MOHAMMADBEIGI, Zahra SAFAYI DELOUYI, Nima MOHAMMADZADEH, Arash ALA’ALMOHADESIN, Keyvan TAHERI, Elahe EDALATI, Mansour SEDIGHI, Abed ZAHEDI BIALVAEI

**Affiliations:** 1 Department of Microbiology and Immunology, Qazvin University of Medical Sciences, Qazvin Iran; 2 Department of Microbiology, School of Basic Sciences, Qom Branch, Islamic Azad University, Qom Iran; 3 Department of Microbiology, School of Medicine, Iran University of Medical Sciences, Tehran Iran; 4 Department of Biology, Damghan Branch, Islamic Azad University, Damghan Iran; 5 Department of Microbiology, Kerman Branch, Islamic Azad University, Kerman Iran; 6 Azarbaijan-Gharbi Regional Blood Transfusion Center, Urmia Iran

**Keywords:** *Clostridium difficile*, prevalence, *toxin A*, toxin B, antimicrobial resistance, Iran

## Abstract

**Background/aim:**

*Clostridium difficile* is a frequent cause of nosocomial infections and has become a major public health concern in developed nations. In the present study, the prevalence and antimicrobial susceptibility pattern of toxigenic *C. difficile* strains isolated in Iran were investigated.

**Materials and methods:**

Between June 2016 and May 2017, 2947 inpatient fecal samples were taken from symptomatic adult hospitalized patients in different units of 32 care facilities in Tehran, Iran. *C. difﬁcile* strains were identified by microbiological/biochemical methods. Susceptibility to 20 antimicrobials was measured by E-test method. Toxin-speciﬁc immunoassays and cytotoxicity assays were used to determine in vitro toxin production.

**Results:**

Out of 2947 fecal samples, 538 (18.25%) *C. difﬁcile* isolates were obtained among those with suspected CDI. In E-test method, all *C. difﬁcile* isolates were susceptible to fidaxomicin, vancomycin, amoxicillin/clavulanate, and meropenem and were resistant to penicillin G. The prevalence of multidrug resistant *C. difficile* was 69.33% (373/538). Among 538* C. difﬁcile*, 147 (27.32%), 169 (31.41%), and 222 (41.26%) isolates were TcdA+/TcdB+, TcdA-/TcdB+, and TcdA-/TcdB-, respectively.

**Conclusion:**

The results evidently support the hypothesis of a probable role of toxigenic strains of *C. difficile* in developing gastrointestinal complaints in patients with diarrhea.

## 1. Introduction

*Clostridium difﬁcile* is an anaerobic, fermentative, spore-forming, and gram-positive *bacillus* (1). The spectrum of *C. difﬁcile*-associated disease (CDAD) varies from mild diarrhea to severe life-threatening colitis, and it may lead to toxic megacolon, perforation, sepsis, and even death (2,3). *C. difﬁcile* infection (CDI) is one of the most important nosocomial infections, and in the past decade, its incidence has increased noticeably worldwide (4). The clinical features of CDI are mediated by cellular exotoxins secreted into the colonic environment during bacterial growth. Toxigenic *C. difﬁcile* strains are responsible for nearly all cases of pseudomembranous colitis (PMC) and for 15% to 25% of cases of antibiotic-associated diarrhea (AAD). *C. difﬁcile* is also considered the most common cause of antibiotic-associated colitis (AAC) in developed countries (1,3,5,6). In the last 20 years, *C. difﬁcile* has also emerged as a main cause of nosocomial diarrhea in adult patients and has been responsible for large outbreaks in hospital settings (7,8). For hospitalized patients, the overall incidence of *C. difﬁcile*-associated diseases has been found to vary widely, from 0.1 to 2 per 100 patient admissions (7,9,10). In many hospitals, *C. difﬁcile* is the most frequently isolated enteropathogen (11).

The pathogenicity of this bacterium is determined by the production of two major toxins: enterotoxin A (Toxn A or TcdA; 308 kDa) and cytotoxin B (Toxin B or TcdB; 270 kDa), which are the major virulence factors of this microorganism and which are encoded by two separated genes, called *tcdA* and *tcdB*, located in close vicinity on the chromosome (12,13). Together with three additional genes (*tcd*C, *tcd*D, and *tcd*E), they form a 19.6-kb chromosomal pathogenicity locus named PaLoc (11,14). These toxins ultimately mediate diarrhea and colitis (11).

Toxin A can cause accumulation of fluid and mucosal damage in several animal models such as rabbit ileal and colonic loops, hamster cecal segments, as well as in mouse and rat intestines (15,16). Toxin B has no enterotoxic activity, but it is a stronger cytotoxin than toxin A in tissue culture line by nearly one thousand fold (16). Toxins A and B both disrupt the actin cytoskeleton of gut epithelial cells by the UDP-glucose-dependent glucosylation of proteins from the Rho and Ras subfamilies (17,18). Some strains of *C. difﬁcile* also produce an additional toxin, actin-speciﬁc ADP-ribosyltransferase (binary toxin) called CDT, which was ﬁrst explained by Popoff et al. in 1988 (19). The binary toxin CDT is unrelated to the well-characterized Toxins A and B, and the signiﬁcance of this is uncertain (11). Additionally, antibiotic exposure is considered to be one of the main risk factors for CDAD (20). Indeed, previous exposure to antibiotics, which can disrupt the intestinal normal ﬂora, is the major risk factor for CDI (21,22). Extended-spectrum cephalosporins and clindamycin are the antibiotics most commonly implicated in CDI (4). Many antimicrobials are traded as over-the-counter drugs in Iran. When coupled with a general lack of information regarding the correct use of antimicrobials in the community, misuse is predictable (20,23). Of concern is the fact that toxigenic isolates are capable of converting toxin-negative strains to toxin-positive strains via horizontal transfer of the pathogenicity locus and that transfer of mobile genetic elements carried out antimicrobial resistance genes has been revealed in vitro (24,25). Little is known about the prevalence and antimicrobial susceptibility pattern of toxigenic *C. difficile *isolates in Iran. Therefore, the present study was designed to determine the prevalence of *C. difficile* containing the TcdA and TcdB toxins among strains isolated in Iran and second to characterize the antimicrobial susceptibility profile of these isolates.

## 2. Materials and methods

### 2.1. Deﬁnitions

A CDI case was deﬁned as a patient with diarrhea and a positive laboratory assay for *C. difﬁcile* toxin A and/or B in stools (26). A CDI was classiﬁed as severe if a patient also fulﬁlled at least one of the following criteria: (i) polynuclear neutrophil count ≥20,000/mm3; or (ii) concentration of serum albumin <35 g/L. The patients were classiﬁed into three groups, based on the severity of the underlying infection, according to the McCabe score: (A) no fatal disease, (B) fatal disease in the following 5 years, or (C) fatal disease in the following year (27). The nosocomial infection (HA-CDI) was diagnosed in patients who developed diarrhea at least 72 h after admission or within 2 months of the last discharge provided that they were not residents in a long-term facility, and they tested positive for CDI (28). Only one episode/patient was involved in the study. An episode was designated as a recurrence when it occurred within 8 weeks of the onset of a previous episode (26).

### 2.2. Design of the study and sample collection 

Two thousand nine hundred forty-seven inpatient fecal specimens (one specimen per patient) submitted for routine CDI testing from June 2016 to April 2017 inclusive were included in the study. All strains were recovered from patients hospitalized in 32 different care facilities in Tehran, Iran, or its surroundings. Only diarrheal fecal specimens were included in the study. Samples from children younger than 2 years old were excluded. The samples were tested either on the collection day or stored at 2–8 °C for testing within ≤48 h, and then frozen at −20 °C or −70 °C for further toxicity assay.

### 2.3. Culture and identiﬁcation of C. difﬁcile isolates

Fecal samples collected from patients suspected to be infected with *C. difﬁcile* were first treated with alcohol, and then the mixture was inoculated on cycloserine-cefoxitin fructose egg yolk agar (CCFA) plates prepared with a *C. difficile* agar base and selective supplement (Oxoid, Basingstoke, UK). The plates were incubated in an anaerobic chamber with GENbag Anaer (bioMérieux, Marcy l’Etoile, France) at 37 °C for 72 h. The isolates were confirmed as *C. difﬁcile* by the characteristic morphology of the colonies, horse odor, green-yellow ﬂuorescence under UV light (365 nm), gram staining, and the API 20A biochemical test (bioMe´rieux). All isolates were stored at −70 °C in brain–heart infusion broth with 10% glycerol until subsequent analyses.

### 2.4. Antimicrobial susceptibility testing 

Minimal inhibitory concentrations (MICs) of 20 antibiotics (chloramphenicol, penicillin G, fidaxomicin, vancomycin, metronidazole, rifampin, clindamycin, tetracycline, fusidic acid, linezolid, erythromycin, trimethoprim/sulphamethoxazole, bacitracin, ciprofloxacin, piperacillin/tazobactam, amoxicillin/clavulanate, moxifloxacin, gatifloxacin, levofloxacin, and meropenem) were evaluated for all isolated strains of *C. difﬁcile* using E-test strips (AB Biodisk, Durham, NC, USA). Testing and interpretation of MICs results (clinical breakpoint determination) followed the recommended guidelines of Clinical and Laboratory Standards Institute (CLSI) and European Committee on Antimicrobial Susceptibility Testing (EUCA ST). A strain with resistance to ≥3 antimicrobial classes was defined as multidrug resistance (MDR). Strains of *C. difficile* ATCC 700057 and *Escherichia coli* ATCC 25922 were included in each run as controls.

### 2.5. Detection of C. difﬁcile toxins

Toxin-speciﬁc immunoassays and cytotoxicity assays were used to determine in vitro toxin production. A single colony of *C. difﬁcile* isolates was inoculated into brain heart infusion (BHI) broth (Oxoid, Basingstoke, UK) and were cultured anaerobically for 48 h. Broth cultures were centrifuged at 4000 × *g* for 10 min, after which the supernatants were ﬁltered through 0.2-lM Acrodisc syringe ﬁlters (Pall Corp., Portsmouth, UK) and stored at –20 °C for up to 3 months before analysis of toxin production. Toxin A (TcdA) was detected using the *C. difﬁcile* Tox A ELISA (Tech Laboratory, Blacksburg, VA, USA) and an immune-chromatography assay by antitoxin A antibody labelled with latex, the *C. difﬁcile* toxin A test (Oxoid), according to the manufacturer’s instructions. Additionally, the immunoenzymic assay *C. difﬁcile* Tox A/B test (Tech Laboratory) was used for detection of either TcdA and/or TcdB toxins. The procedures were conducted according to the manufacturer’s instructions. Toxin B was detected by a cell culture cytotoxin assay on the McCoy cell line. In brief, ten-fold serial dilutions of ﬁltered bacterial supernatants were added in duplicate to McCoy cells and incubated for 24 h. *C. difﬁcile* VPI-10463 was used as a positive control. The cytopathic effect (CPE) was surveyed by inverse microscopy. If this CPE could be neutralized by polyclonal antiserum to *C. difﬁcile* (*C. difﬁcile* TOX-B Test; TechLab), the test was considered positive.

### 2.6. Statistical analysis

Statistical analysis was performed with SPSS software, version 16.0 for Windows (SPSS Inc., Chicago, IL, USA). P-value <0.05 was considered statistically significant.

## 3. Results

During the 12 months of the study period, from 2947 fecal samples taken from symptomatic adult hospitalized patients in different units of the 32 designated hospitals, 538 (18.25%) *C. difﬁcile* isolates were obtained among those with suspected CDI, which confirmed as HI-CDI agent. According to classification of patients with CDI as mentioned above, 486 were in class A, 35 were in class B, and 17 were in class C. These 538 *C. difﬁcile* isolates were analyzed for TcdA and/or TcdB toxins. Using the *C. difﬁcile* ToxA ELISA, the *C. difﬁcile *toxin A test, the ToxA/B test, and the TcdB cytotoxicity testing on McCoy cells, 147 (27.32%) isolates produced detectable toxin A and toxin B (TcdA+/TcdB+). One hundred sixty-nine (31.41%) isolates were toxin B-positive but toxin A-negative (TcdA-/TcdB+), because TcdA could not be detected using the commercial latex test for TcdA, but a CPE on the McCoy cell line was observed. The TOX A/B tests gave positive results for all 169 A-B+ strains. The remaining 222 (41.26%) *C. difﬁcile* isolates were negative for toxin production (TcdA-/TcdB-) because all tests gave negative results. Furthermore, all 316 (58.74%) toxin-positive isolates (TcdA+/TcdB+ and TcdA-/TcdB+) induced a CPE when investigated using the cell culture cytotoxicity assay (Table 1). The CPE observed for the 169 isolates that were negative in the toxin A ELISA was atypical, demonstrating complete cell rounding of the McCoy cell line body with no cytoplasmic extensions. In total, among 538 *C. difﬁcile* isolates, 222 (41.26%) were nontoxigenic and 316 (58.74%) isolates were toxin producer by conducted test. The highest and lowest incidence of toxin positive strains in hospital wards was related to ICU/CCU (n=135; 42.72%) and Trauma center (n = 4; 1.26%). Distribution of toxin positive strains in different hospital units are shown in Table 2. The antimicrobial susceptibility patterns of 538 *C. difficile* isolates, measured by MIC-Etest method, are presented in Table 3. All *C. difﬁcile* isolates were susceptible to fidaxomicin, vancomycin, amoxicillin/clavulanate and meropenem and were resistant to Penicillin G. The antimicrobial resistance rates were distinctly higher for fusidic acid, ciprofloxacin, clindamycin, levofloxacin and erythromycin than PIP-TAZ, metronidazole, rifampin, moxifloxacin, gatifloxacin, vancomycin and tetracycline (P < 0.05). The antimicrobial resistance rates were meaningfully higher for penicillin G, trimethoprim/sulphamethoxazole, bacitracin, ciprofloxacin, erythromycin, levofloxacin, clindamycin than for fusidic acid, tetracycline, gatifloxacin, moxifloxacin, chloramphenicol, metronidazole, rifampin, piperacillin/tazobactam, linezolid, fidaxomicin, vancomycin, amoxicillin/clavulanate, and meropenem (P < 0.05). The prevalence of MDR, as defined by resistance to ≥3 antimicrobials tested, in all *C. difﬁcile* isolates was 69.33% (373/538). Exactly, 118 (80.27%), 121 (71.59%), and 134 (60.36%) of A+/B+, A-/B+, and A-/B- *C. difﬁcile* strains were MDR, respectively. The prevalence of MDR was significantly higher in toxigenic* C. difﬁcile* strains (239/316; 75.63%) in comparison to nontoxigenic strains (134/222; 60.36%) (P < 0.05).

**Table 1:** Summary of results for detection of toxigenic and nontoxigenic Clostridium difficile strains.

	No. (%) ofsamples	Toxigenicstatus	ToxinA ELISA	ToxinA+B ELISA	Cell culturecytotoxicity assay
538	147 (27.32%)	A+/B+	+	+	+
	169 (31.41%)	A-/B+	-	+	+*
	222 (41.26%)	A-/B-	-	-	-

*Variant cytopathic effect observed

**Table 2:** Distribution of toxin-positive and toxin-negative strains in different hospital units.

Wards	No. of C. difﬁcilestrains	No. of toxigenicstrains (%)	No. of nontoxigenicstrains (%)
ICU/CCU	211	118 (37.34%)	93 (41.89%)
General surgery	121	83 (26.26%)	38 (17.12)
Transplant	78	37 (11.70)	41 (18.47)
Internal medicine	51	32 (10.12%)	19 (8.56)
Burn unit	27	11 (3.48%)	16 (7.21)
Cardiology	15	9 (2.84%)	6 (2.7)
Oncology	9	9 (2.84%)	0 (0)
Orthopedic	13	8 (2.53%)	5 (2.25)
Urology	6	6 (1.89%)	0 (0)
Trauma center	7	3 (0.94)	4 (1.8)
Total	538	316 (100)	222 (100)

**Table 3:** Distribution of antibiotic susceptibility patterns of C. difficile isolates based on toxin production.

Antibiotics	Toxigenic strains (n = 316)						Nontoxigenic strains (n = 222)		
	A+/B+ (n = 147)			A-/B+ (n = 169)			A-/B-		
	S	I	R	S	I	R	S	I	R
	N (%)	N (%)	N (%)	N (%)	N (%)	N (%)	N (%)	N (%)	N (%)
Chloramphenicol	79 (53.74)	35 (23.8)	33 (22.44)	60 (35.5)	74 (43.78)	35 (20.71)	121 (54.5)	62 (27.92)	29 (13.06)
Penicillin G	0 (0.00)	0 (0.00)	147 (100)	0 (0.00)	2 (1.18)	167 (98.82)	0 (0.00)	5 (2.25)	217 (97.75)
Fidaxomicin	147 (100)	0 (0.00)	0 (0.00)	169 (100)	0 (0.00)	0 (0.00)	222 (100)	0 (0.00)	0 (0.00)
Vancomycin	147 (100)	0 (0.00)	0 (0.00)	169 (100)	0 (0.00)	0 (0.00)	222 (100)	0 (0.00)	0 (0.00)
Metronidazole	127 (86.39)	8 (5.44)	12 (8.16)	143 (84.61)	6 (3.55)	20 (11.83)	214 (96.39)	8 (3.6)	0 (0.00)
Rifampin	127 (86.39)	11 (7.48)	9 (6.12)	151 (89.34)	7 (4.14)	11 (6.5)	215 (96.84)	7 (3.15)	0 (0.00)
Clindamycin	20 (13.6)	21 (14.28)	106 (72.1)	28 (16.56)	32 (18.93)	109 (64.49)	41 (18.46)	25 (11.26)	156 (70.27)
Tetracycline	70 (47.61)	13 (8.84)	64 (43.53)	66 (39.05)	34 (20.11)	69 (40.82)	85 (38.28)	30 (13.51)	107 (63.31)
Fusidic acid	52 (35.37)	23 (15.64)	72 (48.97)	86 (50.88)	16 (9.46)	68 (40.23)	157 (70.72)	22 (9.9)	43 (19.36)
Linezolid	142 (96.59)	2 (1.36)	3 (2.04)	168 (99.4)	1 (0.6)	0 (0.00)	219 (98.64)	1 (0.45)	2 (0.9)
Erythromycin	35 (23.8)	0 (0.00)	112 (76.2)	54 (31.95)	0 (0.00)	115 (68.05)	46 (20.72)	0 (0.00)	176 (79.28)
Ciprofloxacin	18 (12.24)	4 (2.72)	125 (85.03)	22 (13.01)	6 (3.55)	141 (83.43)	29 (13.06)	0 (0.00)	193 (86.94)
Piperacillin/tazobactam	130 (88.43)	11 (7.48)	6 (4.08)	154 (91.12)	6 (3.55)	9 (5.32)	207 (93.24)	7 (3.15)	8 (3.6)
Amoxicillin/clavulanate	147 (100)	0 (0.00)	0 (0.00)	169 (100)	0 (0.00)	0 (0.00)	222 (100)	0 (0.00)	0 (0.00)
Trimethoprim/sulphamethoxazole	14 (9.52)	0 (0.00)	133 (90.47)	7 (4.14)	0 (0.00)	162 (95.85)	1 (0.45)	0 (0.00)	221 (99.55)
Moxifloxacin	94 (63.94)	10 (6.8)	43 (29.25)	115 (68.04)	17 (10.05)	37 (21.89)	164 (73.87)	6 (2.7)	52 (23.42)
Gatifloxacin	93 (63.26)	2 (1.36)	52 (35.37)	119 (70.41)	5 (2.95)	45 (26.62)	178 (80.18)	4 (1.8)	40 (18.01)
Levofloxacin	29 (19.72)	16 (10.88)	102 (69.38)	31 (18.34)	7 (4.14)	131 (77.51)	39 (17.56)	11 (4.95)	178 (80.18)
Bacitracin	7 (4.76)	0 (0.00)	140 (92.23)	21 (12.42)	0 (0.00)	148 (87.57)	13 (5.85)	0 (0.00)	209 (94.14)
Meropenem	147 (100)	0 (0.00)	0 (0.00)	169 (100)	0 (0.00)	0 (0.00)	222 (100)	0 (0.00)	0 (0.00)
MDR	118 (80.27)			121 (71.6)			134 (60.36)		
	239 (75.93)								

S: susceptible, I: intermediate, R: resistant.

## 4. Discussion

*Clostridium difficile* is a microorganism that can be found in most individuals without causing symptoms, but in some people it can cause a severe diarrhea and colitis. The bacterium is typically acquired from the hospitals, as environmental contamination is common and healthcare personnel may carry it in their hands, or on contaminated devices (3,29). CDI is an increasing public health concern worldwide and is the primary cause of intestinal infection associated to antimicrobial treatment (30). Due to being rapid, cost-effective, and easy performance of testing; ELISAs, immune-chromatography, immunoenzymic, and cytotoxicity (cell culture) assays are now used most frequently by clinical laboratories for diagnosis of *C. difficile* infection (31). The frequency of *C. difficile* infection among the patients suffering from CDAD is different throughout the world. The global prevalence of CDAD is 0.9% and 2% in the general population and ICU patients, respectively (32). A comparable pattern is observed in Asia (3%) and Europe (1%) (32). Additionally, investigations have revealed that 3.6%, 3.3%, 3.3%, 2.4%, 0.9%, and 20% of CDAD in hospitalized patients of the USA, UK, Canada, China, France, and Taiwan are associated to *C. difficile* infection, respectively (33–35). The prevalence of CDI or CDAD has been less studied in Iran. In our study, *C. difficile* was responsible for 18.25% of the suspected patients with nosocomial diarrhea. These cases came from ICU/CCU, general surgery, transplant, internal medicine, burn unit, cardiology, oncology, orthopedic, urology ,and trauma center.

According to a previous study in Iran, *C. difficile* was isolated from 5.3% of patients with gastrointestinal complaints, 6.1% of patients with nosocomial diarrhea and 4% of children with diarrhea (31,36,37). Zarandi et al. (2017) indicated that the frequency of CDI was 21% among diarrheal samples from ICU (38). In another research the prevalence of CDI was near 20% in hospitalized patients (39), which is similar to our findings. The frequency of CDI was reported 4.9% for Turkish patients but this amount was 18% for Canadian patients with nosocomial diarrhea, which was consistent with our results (40,41). In the survey conducted by Langley et al. (2002), *C. difﬁcile* (with 32% prevalence) was one of the most common pathogens in nosocomial diarrheal episodes (42). In a study carried out by Gursoy et al. (2007), the total prevalence of *C. difﬁcile* was 27.7% (43). In Brazil, *C. difficile* was responsible for 5.5% of hospitalized children with severe diarrhea and in Argentina, *C. difficile-*positive specimens were identified in 38.5% of symptomatic patients (16,44).

Effective treatment of CDI is frequently based on common sensitivity reports for the strains in each country. There are a few reports about the prevalence of MDR phenotype among the clinical isolates in some countries (45,46). Shayganmehr et al. (2015) reported high resistance rate of *C. difficile* isolates to ciprofloxacin (97%), and low resistance rate to metronidazole (5%), which is similar to our results (47). High level fluoroquinolone-resistant *C. difficile *strains was previously reported by Nore´n et al. (2010), who investigated resistance frequency of isolates to moxifloxacin (23%), levofloxacin (100%), and ciprofloxacin (100%) (48). Data from the current study showed that 8.16% of our strains were resistant to metronidazole that was higher than the overall reported rate of resistance from other studies (49–51). This resistance level probably was caused by indiscriminate use of metronidazole in CDI and also in other common cases of protozoal infections in Iran. In the present study, the analysis of the antimicrobial resistance phenotypes among the 538* C. difficile* isolates showed 89 strains with single drug resistance (16.54%), 76 strains with double drug resistance (14.12%), and 373 isolates with triple antibacterial resistance or MDR phenotypes (69.33%). All the strains with resistance phenotypes to metronidazole belonged to the triple drug resistance groups. In a study in Italy, out of 316 *C. difficile* clinical isolates, 12 (3.7%) were resistant to only one antibiotic, 54 (17%) to two antibiotics, and 82 (26%) to at least three antibiotics (MDR); however, reduced susceptibility to metronidazole was not found among the MDR strains (46). In a similar research in Kuwait, there was no resistance to metronidazole among* C. difficile* isolates, while MDR phenotype was observed in 75.3% of isolates and double and triple resistance phenotypes were identified in 11% and 38.3% of the isolates, respectively (52). Most of the MDR strains in our study were toxigenic (75.93%). Concurrent resistance to the tested antibiotics was significant in the tcdA+/B+ toxigenic group. These findings cast new light to the association between toxin producer strains and resistance phenotype in *C. difficile*. This relationship was previously reported by other researchers (46,53,54). It has been demonstrated that toxigenic *C. difficile* isolates are resistant to broad spectrum antimicrobial drugs, such as β-lactams, flouroquinolones, and clindamycin (55). It has been also shown that mean intake of several beta-lactams and fluoroquinolones was higher in affected hospitals with the resistant-toxigenic *C. difficile* strains, which suggests the involvement of widespread antibiotic prescription in selection of toxigenic isolates in these hospitals (56). The relationship between toxigenicity and resistance phenotype of the *C. difficile* strains was also supported by a recent finding about cotransfer of *C. difficile* pathogenicity locus, encoding the two noted toxins, with conjugative transposons encoding resistance to several antibiotics (24).

The prevalence of toxigenic *C. difﬁcile* nosocomial diarrhea varied in different studies, but there are a limited number of studies in this field in Iran. Sadeghifard et al. (2010) analyzed a total of 942 stool samples from Iranian patients with nosocomial diarrhea. They showed that 57 samples (6.1%) were positive for toxigenic *C. difficile* (57). Among the strains investigated in this study, 27.3% were A+/B+, 31.4% were A-/B+, and 41.2% were A-/B-. In the study conducted by Pituch et al. (2006), 43%, 45.5 %, and 8.9 % were A+/B+, A-/B+, and A-/B-, respectively (12). In their previous study (in the period 1999–2001 from patients with CDAD) among 33 *C. difficile* strains, 45% were A+/B+ but 55% were A-/B+ (58). The ﬁndings of the present study conﬁrmed the high prevalence of toxigenic isolates in our hospitals. Rezazadeh Zarandi et al. (2017) demonstrated that 49.57% of *C. difficile *isolates did not carry tcdA and tcdB genes. Interestingly, 77.5% of the total isolates belonged to nontoxigenic type (A-/B- and A+/B-) and 22.5% were toxin-producing (A+/B+) (38). They demonstrated that nontoxigenic type A-/B- was the most prevalent type (38). Similar to our results, several studies have shown that the nontoxin production type (A-/B-) is the most prevalent (42%–50%) in clinical data (59,60). In some studies, they were regarded as pathogenic while as nonpathogenic in others (59,61). In this study, they are considered nonpathogenic. Toxin production type, A+/B+ which is clearly associated with CDAD, has up to 71.6% prevalence among *C. difficile *toxin production types globally (38), while the A-/B+ type was prevalent in our isolates, although its prevalence was lower than that of nontoxigenic strains in Iran and other parts of the world (38). In total, nontoxigenic isolates are prevalent in clinical samples.

To conclude, this study indicates that *C. difficile* might be an important enteric pathogen in patients in Iranian hospitals. As long as toxigenic *C. difﬁcile* strains are recognized as the primary cause of severe diarrheal disease, the best management approach is to further our understanding of this opportunistic pathogen and improve the diagnostic methods. Further study of the epidemiology and microbiology of CDI in this region is required to explore some apparent differences. As a consequence, it seems necessary to investigate the mechanisms involved in the infection and pathogenesis of this organism such as production of various toxins. Our results showed an association between the coexistence of tcdA+/tcdB+ genes and MDR phenotypes among the clinical isolates of *C. difficile*. This finding emphasizes the need for continuous monitoring of antimicrobial susceptibility patterns among the pathogenic strains for prevention of the occurrence of eradication failure in the infected patients.

**Acknowledgment**

The authors would like to thank Saadat Pirouzi for statically analysis and technical assistance in the present study.

## References

[ref0] (2002). High prevalence of toxin A-negative toxin B-positive Clostridium difficile in hospitalized patients with gastrointestinal disease. Diagn Microbiol Infect Dis.

[ref1] (2012). Clostridium difficile: a problem of concern in developed countries and still a mystery in Latin America. J Med Microbiol.

[ref2] (2018). Clostridium difficile infection: a review. Rev Med Microbiol.

[ref3] (2013). Epidemiology of Clostridium difficile infections in a tertiary‐care hospital in Korea. Clin Microbiol Infect.

[ref4] (1998). Increasing hospitalization and death possibly due to Clostridium difficile diarrheal disease. Emerg Infect Dis.

[ref5] (2002). Antibiotic-associated diarrhea. N Engl J Med.

[ref6] (1992). Nosocomial infection with Clostridium difficile investigated by pyrolysis mass spectrometry. J Med Microbiol.

[ref7] (1989). Nosocomial acquisition of Clostridium difficile infection. N Engl J Med.

[ref8] (1995). Isolation of Clostridium difficile at a university hospital: a two-year study. Clin Infect Dis.

[ref9] (1982). Ten years of prospective Clostridium difficile-associated disease surveillance and treatment at the Minneapolis VA Medical Center,. Hosp Epidemiol.

[ref10] (2004). Prevalence and characterization of a binary toxin (actin-specific ADP-ribosyltransferase) from Clostridium difficile. J Clin Microbiol.

[ref11] (2006). Prevalence and association of PCR ribotypes of Clostridium difficile isolated from symptomatic patients from Warsaw with macrolide-lincosamide-streptogramin B (MLSB) type resistance. J Med Microbiol.

[ref12] (2005). Clostridium difficile toxins: mechanism of action and role in disease. Clin Microbiol Rev.

[ref13] (2002). Prevalence and genetic characterization of toxin A variant strains of Clostridium difficile among adults and children with diarrhea in France. J Clin Microbiol.

[ref14] (1985). Effects of Clostridium difficile toxins given intragastrically to animals. Infect Immun.

[ref15] (2003). Prevalence of Clostridium spp. and Clostridium difficile in children with acute diarrhea in Sao Paulo city Brazil. Mem Inst Oswaldo Cruz.

[ref16] (1995). Glucosylation of Rho proteins by Clostridium difficile toxin B. Nature.

[ref17] (1995). The enterotoxin from Clostridium difficile (ToxA) monoglucosylates the Rho proteins. J Biol Chem.

[ref18] (2004). Distribution of Clostridium difficile variant toxinotypes and strains with binary toxin genes among clinical isolates in an American hospital. J Med Microbiol.

[ref19] (2017). Antimicrobial susceptibility of Clostridium difficile isolated in Thailand. Antimicrob Resist Infect Control.

[ref20] (2010). The changing epidemiology of Clostridium difficile infections. Clin Microbiol Rev.

[ref21] (2007). Clostridium difficile: emergence of hypervirulence and fluoroquinolone resistance. Infection.

[ref22] (2012). Antibiotics Smart Use: a workable model for promoting the rational use of medicines in Thailand. Bull World Health Organ.

[ref23] (2013). Horizontal gene transfer converts non-toxigenic Clostridium difficile strains into toxin producers. Nat Commun.

[ref24] (2013). Phage ϕC2 mediates transduction of Tn6215, encoding erythromycin resistance, between Clostridium difficile strains. MBio.

[ref25] (2006). Emergence of Clostridium difficile‐associated disease in North America. Clin Microbiol Infect.

[ref26] (1962). Gram-negative bacteremia: I. Etiology and ecology. Arch Intern Med.

[ref27] (2010). Hypervirulent Clostridium difficile strains in hospitalized patients. Canada. Emerg Infect Dis.

[ref28] (1996). Clostridium difficile infection. Crit Rev Clin Lab Sci.

[ref29] (2017). Molecular epidemiology of Clostridium difficile infection in hospitalized patients in Eastern China. J Clin Microbiol.

[ref30] (2011). Frequency of Clostridium difficile among patients with gastrointestinal complaints. Gastroenterol Hepatol Bed Bench.

[ref31] (2015). Prevalence and clinical outcomes of Clostridium difficile infection in the intensive care unit: a systematic review and meta-analysis. Open Forum Infect Dis.

[ref32] (2014). ICU-Onset Clostridium difficile infection in a university hospital in China: a prospective cohort study. PLoS One.

[ref33] (2015). Impact of toxigenic Clostridium difficile colonization on the risk of subsequent C. difficile infection in intensive care unit patients. Infect Control Hosp Epidemiol.

[ref34] (2016). Clostridium difficile infections in medical intensive care units of a medical center in Southern Taiwan: variable seasonality and disease severity. PLoS One.

[ref35] (2005). Prevalence of Clostridium difficile-associated diarrhea in hospitalized patients with nosocomial diarrhea. Iran J Public Health.

[ref36] (2009). Toxigenic Clostridium difficile colonization in children. J Pediatr Infect Dis.

[ref37] (2017). Frequency of antibiotic associated diarrhea caused by Clostridium difficile among hospitalized patients in intensive care unit. Gastroenterol Hepatol Bed Bench.

[ref38] (2012). Clostridium difficile infection in an Iranian hospital. MC Res Notes.

[ref39] (1996). Clostridium difficile acquisition rate and its role in nosocomial diarrhoea at a university hospital in Turkey. Eur J Epidemiol.

[ref40] (2002). Canadian Nosocomial Infection Surveillance Program. Morbidity, mortality, and healthcare burden of nosocomial Clostridium difficile-associated diarrhea in Canadian hospitals. Infect Control Hosp Epidemiol.

[ref41] (2002). The role of Clostridium difficile and viruses as causes of nosocomial diarrhea in children. Infect Control Hosp Epidemiol.

[ref42] (2007). Clostridium difficile infection frequency in patients with nosocomial infections or using antibiotics. Hepatogastroenterology.

[ref43] (2003). Clostridium difficile-associated diarrhea from a general hospital in Argentina. Anaerobe.

[ref44] (1982). Investigation of an outbreak of antibiotic-associated colitis by various typing methods. J Clin Microbiol.

[ref45] (2011). Multidrug resistance in European Clostridium difficile clinical isolates. J Antimicrob Chemother.

[ref46] (2015). Clostridium difficile genotype with emergence of multidrug-resistant strains conferring metronidazole resistant phenotype. Iran Biomed J.

[ref47] (2010). In vitro susceptibility to 17 antimicrobials of clinical Clostridium difficile isolates collected in 1993–2007 in Sweden. Clin Microbiol Infect.

[ref48] (2010). van Belkum A. Clostridium difficile infection in Polish pediatric outpatients with inflammatory bowel disease. Eur J Clin Microbiol Infect Dis.

[ref49] (2007). Distribution and antimicrobial susceptibility patterns of Clostridium difficile PCR ribotypes in English hospitals. Euro Surveill.

[ref50] (2007). Molecular characterization and antimicrobial susceptibility patterns of Clostridium difficile strains isolated from hospitals in south-east Scotland. J Med Microbio.

[ref51] (2002). In vitro activity of 15 antimicrobial agents against clinical isolates of Clostridium difficile in Kuwait. Int J Antimicrob Agents.

[ref52] (2012). Multidrug-resistant North American pulsotype 2 Clostridium difficile was the predominant toxigenic hospital-acquired strain in the province
of Manitoba, Canada, in 2006–2007. J Med Microbiol.

[ref53] (1999). Epidemics of diarrhea caused by a clindamycin-resistant strain
of Clostridium difficile in four hospitals. N Engl J Med.

[ref54] (2008). New advances in the treatment of Clostridium difficile infection (CDI). Ther Clin Risk Manag.

[ref55] (2010). Is high consumption of antibiotics associated with Clostridium difficile polymerase Chain Reaction–ribotype 027 infections in. France? Infect Control Hosp Epidemiol.

[ref56] (2010). The incidence of nosocomial toxigenic Clostridium difficile associated diarrhea in Tehran tertiary medical centers. Acta Medica Iranica.

[ref57] (2003). Recent emergence of an epidemic clindamycin-resistant clone of Clostridium difficile among Polish patients with C. difficile-associated diarrhea. J Clin Microbiol.

[ref58] (2013). A clinical and epidemiological review of non-toxigenic Clostridium difficile. Anaerobe.

[ref59] (2002). Clostridium difficile, atopy and wheeze during the first year of life. Pediatr Allergy Immunol.

[ref60] (2004). Isolation of non-toxigenic strains of Clostridium difficile from cases of diarrhea among patients hospitalized in hematology/oncology ward. Pol J Microbiol.

